# Predictors of treatment failure among patients with pulmonary tuberculosis attending public health facilities in Nairobi county

**DOI:** 10.1371/journal.pgph.0004131

**Published:** 2025-05-13

**Authors:** Faith Muthoki Mwanzui, Simon Karanja, Alex Kigundu Muriithi, Herman Owuor Weyenga

**Affiliations:** 1 Kenya Medical Training College, Faculty of Public Heath, Deparment of Health Promotion and Community Health, Nairobi, Kenya; 2 Jomo Kenyatta University of Agriculture and Technology (JKUAT), Kenya, Nairobi, Kenya; 3 Center for Disease Control (CDC), Kenya; University of Embu, KENYA

## Abstract

Tuberculosis (TB) is one of the infectious diseases of public health concern globally. Kenya is ranked 15th among the 22 high TB burden countries worldwide, which collectively contribute to 80% of the world’s TB cases. TB Treatment failure is one of the threats to the control of TB. The research aimed at determining affordable predictors of TB treatment failure in a resource limited setting to inform policy in designing public health interventions that are best suited to the country’s needs. To determine the predictors of treatment failure among patients with sputum smear positive pulmonary TB attending selected public health facilities in Nairobi Count. Data was abstracted and summarized from both patients and their medical records, focusing on socio-demographic, behavioral, and clinical exposure data. Data was collected from 4 Sub-counties, a total of 21 public health facilities with high case load of pulmonary TB were reached. Utilizing an unmatched case-control design, the study enrolled 81 patients diagnosed with TB treatment failure (cases) and 162 patients who were declared cured after completing their anti-TB treatment (controls. Strengthen contact tracing, screening, and documentation of TB treatment failure cases. Conduct further studies to elucidate the association between HIV and TB treatment failure. The factors significantly associated with treatment failure in this study encompassed prior exposure to first-line anti-Tuberculosis drugs, positive sputum smear at 2 months of treatment, and suboptimal adherence to anti-TB treatment. These findings contribute valuable insights into the identification of simple predictors of TB treatment failure such as utilizing sputum microscopy or gene expert testing at 2 months of treatment to detect individuals at risk and strengthen the implementation of DOT and TB treatment failure contact tracing protocol.

## Introduction

Tuberculosis (TB) remains a significant public health concern globally, with a disproportionate burden on developing nations [1]. According to the World Health Organization’s (WHO) Global Report in 2019, TB ranks as the ninth leading cause of death worldwide and is a major contributor to mortality from infectious diseases, surpassing (human immune-deficient virus (HIV)/ Acquired Immunodeficiency Syndrome (AIDS). In 2018, an estimated 10.1 million people contracted TB, with Africa accounting for 76% of the cases, and approximately 1,524,000 TB-related deaths were recorded globally [2].Despite the challenges, the true prevalence of TB treatment failure in Kenya remains unknown. However, there is a high potential for progression to drug-resistant TB, including multidrug- resistant TB (MDR-TB) and extensively drug-resistant TB (XDR-TB) [3]. Factors contributing to this include prolonged exposure to first-line drugs during retreatment, weak health systems, high prevalence of HIV/AIDS among TB patients, and limited access to healthcare, particularly among slum dwellers [4]. This study seeks to fill the gap in literature by identifying predictors of TB treatment failure in public health facilities in Nairobi County.

### Statement of the problem

TB treatment failure poses significant challenges in terms of morbidity, mortality, and its potential to reverse progress made in TB control efforts. It is associated with prolonged infectivity, amplification of drug resistance TB, and strains on healthcare systems, particularly in resource- limited settings like Kenya [5]. Surveillance of TB treatment failure requires robust laboratory networks and trained personnel, resources often lacking in these settings, where the burden of TB is highest, especially in urban slums.

The actual prevalence of TB treatment failure in Kenya is unknown, but its potential for contributing to multidrug-resistant TB (MDR-TB) is alarming, given existing challenges such as weak health systems, missed TB cases, and high HIV/AIDS co-infection rates[6]. This situation is exacerbated by an increase in TB treatment failure cases reported, particularly from urban slums, without sufficient efforts to identify predictors associated with these cases [7]. Lack of intervention leaves infected individuals within communities, increasing the risk of disease transmission. To mitigate the consequences of TB treatment failure, there is an urgent need for cost-effective public health interventions tailored to local contexts. Understanding the predictors of TB treatment failure in Nairobi County is essential for developing targeted interventions and policies to address this challenge.

### General objective

To determine predictors of TB treatment failure among pulmonary tuberculosis patients attending selected Public Health facilities in Nairobi County, Kenya.

### Specific objectives

To identify the socio-demographic characteristics associated with TB treatment failure among pulmonary tuberculosis patients attending selected Public health facilities in Nairobi County, Kenya.

To assess behavioral factors associated with TB treatment failure among pulmonary tuberculosis patients attending selected Public health facilities in Nairobi County, Kenya.

To examine clinical factors associated with treatment failure among pulmonary tuberculosis patients attending selected Public health facilities in Nairobi County, Kenya.

## Materials and methods

The research was conducted in selected public health facilities in Nairobi County, known for their high caseload of pulmonary tuberculosis (TB). Nairobi County comprises 17 Sub-counties, housing a total of 247 public health facilities that provide TB treatment services. Sub-counties identified with a high TB caseload, based on county records, included Langat, Starehe, Mathare, Embakasi East and Embakasi West.

An unmatched case-control study design was employed to identify the predictors of TB treatment failure among pulmonary TB patients receiving care at selected public health facilities in Nairobi County. This design was chosen for its ability to assess associations between multiple risk factors and TB treatment failure by comparing cases with controls. Additionally, this design allowed for the efficient utilization of resources within a limited timeframe. The study utilized patient records and data from the Kenya National TB Register, which serves as the primary repository for disease notification data nationwide.

The study included all surviving TB treatment failure patients confirmed by Gene pert, microscopy or culture testing between 2018 to 2022, as well as selected ordinary sputum smear- positive TB patients from the same sites as the treatment failure patients. Both cases and controls were aged 15 years and above.

A minimum sample size of 243 participants (81 cases and 162 controls) was calculated using the Fleiss formula (Fleiss et al., 1981). The proportion of pulmonary TB cases and controls with smear- positive TB relapse was estimated at 79% and 21%, respectively [7].The minimum odds ratio detectable by this study was set at 3.0, with a two-tailed level of significance (α) of 5% and a study power (1-β) of 80% (β = 0.20).

The sample was independently and randomly selected areas with good ventilation to reduce TB transmission risk. Data confidentiality was ensured, with questionnaires kept secure and accessible only to authorized personnel. Ethical clearance was obtained from the KNH-UON Ethical Review Committee. The research protocol reference number is P946/11/2019. Data was first accessed for research purposes from December 2020. Recruitment of participants to the study started on December 2021 and ended on June 2022.

### Data management and analysis

Data were entered into Microsoft Excel version 2016 for ease of management and transferability to statistical software. Double data entry was conducted daily to minimize errors, and data cleaning was performed prior to analysis. A descriptive analysis was done based on frequency distribution of selected socio-demographic characteristics. Means, standard deviations and quartiles of selected study variables were obtained.

The study performed Bivariate analysis to establish any existing associations between the specific individual predictor variables and the outcome measure variable (TB treatment failure), by odds ratio and chi-square statistic. For categorical variables (nominal data) at 95% confidence interval (CI) and alpha level of significance set at 0.05 was used as measures of association in the analysis of factors associated with treatment failure T-test at the same confidence interval and significance level was used for numerical variables.

### Ethical consideration

Ethical clearance was obtained from the Ministry of Higher Education, Science, and Technology, JKUAT Board of Postgraduate Studies, and the KNH-UON Ethical Review Committee. Permission to collect data was granted by the County TB coordinator and the Ministry of Health research committee. Informed consent was obtained from all participants; the study posed no risks of harm to the participants. The benefits of the study included improved understanding, prevention and treatment of TB treatment failure in future. There were no costs or payment by the participants involved. Data collectors were trained on infection prevention measures. Respirators and surgical masks were provided for interviewers and participants, and interviews were conducted in open

## Results

### Distribution of cases and controls by age

The cases had an average age of 32.4 years (standard deviation = 10.4) and a mean of 31 years (interquartile range = 26.0-37.0). Those in the control group had an average age of 34.7 years (standard deviation = 12.6) and a middle value of 32.0 years (interquartile range = 26.0-40.0). Most of the participants in the study fell within the economically active age range of 15–44 years (Table 1).

**Table 1:** Age Distribution of Cases and Controls by age.

Age In Years	Cases (Frequency, n = 81)	Cases (Percentage, %)	Age In Years	Controls (Frequency, n = 162)	Controls (Percentage, %)
<15	2	0.2	< 15	3	1.8
15-24	19	23.4	15-24	29	17.9
25-34	26	32	25-34	62	38.2
35-44	21	25.9	35-44	38	23.4
45-54	7	8.6	45-54	15	9.2
55-64	4	4.9	55-64	11	6.7
>65	2	2.4	> 65	4	2.4

### Socio-demographic factors associated with TB treatment failure

During treatment, the cases typically traveled approximately twice the distance to the TB treatment facility compared to controls (P < 0.001), a pattern also seen in the duration of travel for both groups (P < 0.001). Living within a 3-kilometer radius of the TB treatment facility significantly reduced the risk of TB treatment failure (P = 0.002). Although cases traveled farther, there was no statistically significant difference in the means of transportation used by cases and controls (P = 0.353). While pre-illness, unemployment did not correlate with TB treatment failure, there was a significant threefold increase in unemployment among patients with TB treatment failure compared to controls (OR=3.31, 95% CI = 1.67-6.65; P < 0.001).Cases were three times more likely to lack formal education (OR=3.0, 95% CI = 1.2-7.9; P = 0.030). However, receiving messages about TB during the last episode of ordinary TB treatment was protective (OR=0.28, 95% CI = 0.16-0.50, P = 0.001), a finding that held true regardless of the participants’ level of formal education (Table 2)

**Table 2:** Social demographic characteristics for cases and controls.

Exposure variable	Cases No	(%)	Controls No %		COR	95% CI *LL- *UL	P-Value
Mean distance (Km) travelled to HP(SD)	10.0	(11.6)	5.5	(8.3)	1.58	0.92-3.99	<0.158
Mean duration (minutes) of travel to HP(SD)	42.	(41.0)	25.	(22.3)	2.56	1.19-4.71	0.001
Mean No. of family members (SD)	3.4	(3.7)	3	(2.7)	0.41	0.27-0.77	0.104
Use of public transport (Yes)	29	(35)	47	(29)	1.36	0.77-2.41	0.353
Travelling < 3km to hospital (Yes)	29	(35)	93	(57.4)	0.41	0.23-0.72	0.002
Marital status (married)	37	(45.7)	93	(57)	0.62	0.36-1.07	0.111
Living with family (Yes)	53	(65.4)	92	(74.7)	0.64	0.36-1.14	0.170
Religion (Christian)	50	(61.7)	11	(70.4)	0.68	0.39-1.19	0.226
Gender (Female)	31	(38.3)	66	(35.8)	0.90	0.52-1.56	0.820
Employment before illness (None)	24	(29.6)	42	(25.9)	1.20	0.67-2.18	0.650
Current employment (None)	65	(80.2)	89	(54.9)	3.31	1.67-6.65	0.001
Type of house lived in (Permanent)	43	(53.1)	83	(51.9)	1.07	0.63-1.83	0.890
Homelessness (Yes)	10	(12.3)	11	(6.8)	1.9	0.78-4.76	0.220
Formal Education (None)	11	(13.6)	8	(5.8)	3.03	1.17-7.85	0.030
Received TB Messages (Yes)							
All participants (Yes)	43	(53.1)	13	(80.2)	0.28	0.16-0.50	<0.001
Education≤ Primary (Yes)	21	(46.7)	59	(72)	0.34	0.16-0.73	0.009
Education ≥ Secondary (Yes)	19	(52.8)	67	(83.8)	0.22	0.09-0.52	0.001

### Behavioral factors associated with TB treatment failure

While a smaller percentage of cases 34 (42.3%) acknowledged ever consuming alcohol compared to controls 81(50%), this variance did not reach statistical significance (OR=0.7,95%, CI = 0.4-1.34; P = 0.28). Similarly, the history of cigarette smoking did not yield a statistically significant difference, despite cases reporting a higher prevalence (OR=1.1,95%CI = 0.6-2.1; P = 0.678). Notably, an equal proportion of cases and controls admitted to substance abuse (Table 3).

**Table 3:** Behavioural Factors associated with TB Treatment Failure.

Exposure	Cases (No, %)	Controls (No, %)	COR	95% CI(LL-UL)	P value
Those who have ever smoked (Yes)	33 (39.5)	52 (31.2)	1.15	0.64 - 2.15	0.678
Smoked in the past 2 years (Yes)	17 (19.5)	36 (21.6)	0.74	0.36 - 1.6	0.534
Living with smoker (Yes)	37 (43.0)	61 (37.2)	1.36	0.71 - 2.14	0.456
Ever consumed alcohol (Yes)	35 (42.3)	81 (50)	0.75	0.44 - 1.34	0.280
Taken alcohol in past 2 years (Yes)	10 (12.4)	18 (11.2)	2.24	0.72 - 7.35	1.118
Any Substance abuse (Yes)	18 (22)	33 (21.7)	1.12	0.53 - 1.92	1.112
Size of the family <3 people (Yes)	30 (54.7)	83 (66.2)	0.61	0.33 - 1.18	0.220
Ever travelled out of Kenya (Yes)	19 (26.1)	28 (20.2)	1.59	0.83 - 3.34	0.372
Cough hygiene (Yes)	46 (61.2)	90 (60.2)	1.13	0.62 - 1.91	0.978
Well ventilated house (Yes)	10 (13.1)	10 (6.4)	2.06	0.84 - 5.21	0.132
Ever imprisoned (Yes)	23 (29.9)	45 (28.2)	1.21	0.64 - 2.25	0.865

### Clinical factors associated with cases and control

Clinical data from the facility’s TB treatment register categorized sputum smear results into four groups based on bacillary load: scanty, 1 + , 2 + , and 3 + . The likelihood of cases having a bacillary load of 2+ or higher was four times greater compared to controls (odds ratio [OR] = 4.1, 95% CI = 1.9-9.8; P = 0.002). Conversely, cases were less likely to have undergone Directly Observed Therapy (DOT) compared to controls (OR = 0.38, 95% CI = 0.21-0.66; P < 0.001). Controls were more prone to being HIV seropositive compared to cases (OR = 0.42, 95% CI = 0.23-0.77; P = 0.007)(Table 4)

**Table 4:** Clinical factors among TB treatment failure cases and controls.

Exposure Variables	Cases (No, %)	Controls (No, %)	COR	95%CI (LL-UL)	P value
Bacillary load (<2+)	69 (84.4)	93 (57.6)	4.19	1.78-9.83	0.002
Sero positive HIV status	19 (23.5)	66 (42.4)	0.43	0.24-0.78	0.007
On ARVs before TB diagnosis	72 (88.7)	87 (55.6)	6.41	1.38-30.12	0.002
DOT recorded	52 (83.8)	155 (95.6)	–	–	0.645
DOT self-recorded	36 (48.4)	115 (71.2)	0.36	0.21-0.66	<0.001
DOT once a week	19 (23.5)	34 (20.9)	1.13	0.55-1.91	1.113
Hospital Admission	17 (20.7)	21 (14.1)	1.65	0.82-3.34	0.230
Known diabetic	4 (4.8)	6 (3.7)	1.5	0.28-8.29	0.869
Chronic chest condition	7 (8.5)	5 (3.08)	2.1	0.53-8.34	0.57

Previous tuberculosis treatment was strongly linked to TB treatment failure (OR = 68.5, 95% CI = 26.91-174.39; P < 0.001). Additionally, cases were two and a half times more likely to lack a BCG scar (OR = 2.5, 95% CI = 1.35-4.63; P = 0.005). Poor adherence to treatment, including missed clinic appointments and doses, was also associated with TB treatment failure (OR = 63.7, 95% CI = 21.01-114.03; P = 0.003) and (OR = 68.5, 95% CI = 1.69-2.67; P = 0.0031) respectively. However, there was no statistically significant difference in reported history of contact with known TB or chronic cough between cases and controls (OR = 1.24, 95% CI = 0.71-2.15; P = 0.536) (Table 5).

**Table 5:** Factors in the past medical history for cases and control.

Exposure Variables	Cases (No,%)	Controls (No, %)	COR	95%CI(LL-UL)	P value
Positive sputum smear past 2 months	36(41.3)	6 (3.7)	40.19	17.8-113.7	0.001
Treated previously for TB	79(96.3)	26 (17.4)	65.43	34.21-78.43	<0.001
BCG Scar absent	28(38.7)	29 (17.6)	2.41	1.38-4.12	0.005
Missed clinic appointment	25(38.8)	3 (15.6)	63.7	21.07-116.1	0.645
Missed doses for >2 weeks	17 (30)	21 (13.8)	4.8	1.61-2.66	<0.0031
DOT in previous treatment	32(39.1)	14 (60.9)	0.59	0.25-1.51	0.400
Contact with known TB case	31(38.7)	56 (34.1)	1.45	0.72-2.24	0.536

### Multivariate analysis logistic

A multivariate analysis was conducted by incorporating variables found to be associated with TB treatment failure at a significance level of p ≤ 0.1 in the bivariate analysis into an unconditional logistic regression model. These variables encompassed the distance from the health facility to the patient’s home, provision of TB messages, HIV status, bacillary load, previous treatment with anti- tuberculosis drugs, a positive sputum smear at 2 months into TB treatment, presence of a Directly Observed Therapy (DOT) observer, lack of formal education, missed clinic appointments, and absence of a BCG scar (Table 6)

**Table 6:** Optimal Model from Unconditional Logistic Regression Analysis on Predictors of TB Treatment Failure.

Term	AOR	95%	C.I.	Z-Statistic	P-Value
On DOT	0.2310	0.0923	0.5783	-3.1298	0.0017
Positive HIV	0.3416	0.1333	0.8756	-2.2366	0.0253
Previous TB treatment	85.0237	29.7063	243.3497	8.2809	0.0001
a positive sputum smear at 2 months of TB treatment	75.0237	19.7063	43.3247	6.2459	0.0021
Missed clinic appointments	65.0237	17.7063	39.1127	4.3421	0.0041

## Discussion

This study aimed at identifying predictors of TB treatment failure in selected public health facilities in Nairobi County, Kenya, so as to address knowledge gaps and inform the development of cost-effective interventions tailored to the country’s needs. The distribution of TB cases across age groups mirrored that of controls and national TB program data for smear-positive TB patients National TB and Leprosy Program (NLTP). However, a noteworthy proportion of cases experienced unemployment following the onset of illness, contrasting with the period before illness when no such disparity was observed. This loss of employment among cases may exacerbate living conditions conducive to TB transmission. Living within three kilometers of the TB treatment facility showed a trend towards protection against TB treatment failure, although statistical significance was not achieved in multivariate analysis.

Formal education has been linked to positive health-seeking behaviors. In this study, having any level of formal education was associated with a reduced risk of being diagnosed with TB treatment failure in the bivariate analysis. Similarly, receiving targeted TB messages was significantly associated with a lower risk of TB treatment failure in the bivariate analysis. However, these associations did not attain statistical significance in the multivariate analysis. The aim of patient education is to enhance compliance and adherence to TB treatment to prevent the emergence of drug resistance. Health education has been shown to improve adherence to anti-TB drugs in randomized clinical trials [8], with similar findings reported in a systematic review of randomized controlled trials on strategies to promote adherence to tuberculosis treatment [9]. The protective effect of receiving TB messages was more pronounced among individuals with secondary and tertiary education levels compared to those with no formal education or primary education, suggesting differences in comprehension levels. Targeting this patient population with relevant messages may help mitigate the risk of TB treatment failure [10] In this study, the majority of behavioral factors examined, did not exhibit any statistical significance in relation to TB treatment failure.

### Clinical predictors of TB treatment failure

In this study, the most significant predictors of TB treatment failure were a history of previous tuberculosis treatment (P < 0.001) and a positive sputum smear at two months of treatment (P < 0.0021). This finding aligns with research conducted in New York [11], a population-based survey across 11 countries [12], and a retrospective study in South Africa. Exposure to first-line anti-TB drugs is believed to favor the selection of drug-resistant strains during treatment [13], leading to the proliferation of resistant strains, which may contribute to treatment failure or relapse after completion of treatment.

Patients who fail or relapse after initial treatment with WHO-recommended first-line drugs face a significantly higher risk of TB treatment failure [14]. A positive sputum smear at two months of TB treatment emerged as a robust predictor of treatment failure in our study, consistent with findings in Peru by Chavez et al. (OR 1.7, p = 0.008). This observation underscores the utility of sputum microscopy as a cost-effective tool for identifying individuals at risk, allowing for early intervention by TB programs.

The first two months of TB treatment are critical for eliminating actively dividing bacilli, with the majority of sputum smear-positive patients turning negative within this period[15]. A positive sputum smear at two months could indicate primary drug resistance or the emergence of mutant strains, particularly in the context of poor adherence [16]. Consequently, TB programs may consider extending the intensive phase of treatment if sputum smear remains positive at two months [17].

Interestingly, a high bacillary load at baseline was not associated with treatment failure in our study, contrary to findings by [18] (p < 0.001). This disparity could be attributed to a higher rate of treatment default among those with a higher bacillary load and the intermittent regimen utilized in Singla’s study [19]. It is worth noting that [20]who employed a treatment regimen similar to ours, did not find a high bacillary load at the start of treatment to be a predictor of treatment failure [21]

Poor adherence to treatment emerged as another significant predictor of treatment failure in our study, consistent with findings by [22] (OR 1.4, p < 0.05), [10] (RR 9.9, p < 0.001), and [11](p < 0.001). Poor adherence can lead to the development of drug resistance, potentially explaining treatment failure. Therefore, program interventions such as Directly Observed Therapy Short Course (DOTS), which promote adherence, should be prioritized for patients with prior treatment history with first-line drugs and a positive sputum smear at two months [23].

In a Case-control study across four European countries, coming into contact with a known tuberculosis case was significantly linked to TB treatment failure [24]. However, in our current study, there was no statistically significant difference in the proportion of cases and controls who had contact with known TB cases or chronic cough (P = 0.536). Some research suggests that multidrug-resistant tuberculosis (MDR-TB) is less transmissible than drug-susceptible [25]. Organisms that become resistant to antimicrobials may lose virulence, allowing the host to survive longer and facilitate the multiplication and spread of drug-resistant strains. A nationwide cohort study in Israel found no secondary cases of MDR-TB [26], and similarly, a study in San Francisco, USA, showed that drug-resistant strains were less likely to cause secondary cases. However, it’s important to note that Beijing strains of M. tuberculosis, which exhibit increased virulence, drug resistance, and transmission risk, do not seem to experience this loss of virulence [27]

Applying these findings to Kenya requires consideration of differences in social, economic, behavioral factors, as well as HIV and AIDS prevalence compared to the settings of the studies mentioned. Congregation and congestion in healthcare settings have been identified as risk factors for tuberculosis [28]. However, in our study, hospital admission was not associated with TB treatment failure (P = 0.230), contrary to several reports of nosocomial transmission of TB [29]. This finding also contrasts with a population-based study in Brazil, where a history of hospital admission within two years before treatment failure diagnosis was significantly linked to an increased risk of TB treatment failure [30]. Although our study found no significant association between hospital admission and treatment failure, the number of cases among household contacts suggests active transmission. Therefore, further research specifically addressing nosocomial TB treatment failure transmission is recommended.

Having a Directly Observed Therapy (DOT) observer during TB treatment was protective and an independent predictor of TB treatment failure. (OR= 0.28; P < 0001). These findings are similar to those in a case control study in Hong Kong [3]. DOT prevents development of drug resistance since the treatment supervisor ensures that patient actually swallows the right dose of TB medicines regularly and for the prescribed period. The current TB pandemic is driven by the HIV/AIDS pandemic. A HIV positive sero-status has also been found to be significantly associated with poor outcome of TB treatment. in several studies such as a case control study Peru [8]and that in four countries in Europe [6], retrospective studies in South Africa, and in the USA [31].1n all these studies a HIV positive status was consistently associated with an increased risk of TB treatment failure. However, a descriptive study in India reported a lower association with HIV [16]. In studies conducted in Africa including that in KwaZulu Natal, South African [32]and the Mozambique study [29] HIV was a major risk factor for TB treatment failure. These findings are in tandem with the global TB treatment failure surveillance data that suggests convergence of TB treatment failure and HIV/AIDS. Some studies indicate that development of rifampicin resistance may be spurred by HIV infection [16,17,18] while in other studies no association was demonstrated between HIV infection and TB treatment failure [4]. Multivariate analysis of the current study indicate that a significantly larger proportion of controls were HIV sero-positive compared to cases (P = 0.0253).

In interpreting these results, it is important to consider factors surrounding TB treatment failure surveillance, epidemiology and control in Kenya that may considerably differ from the other countries. These factors may favors accelerated TB disease progression among the HIV infected patients, selective survival of, and transmission of TB treatment failure among the HIV sero-negative individuals. Many African countries including Kenya are said to be underreporting cases of TB treatment failure[30].Secondly, Kenya has a high prevalence of HIV related TB which is difficult to diagnose due to atypical presentation and lack of appropriate tests to detect smear negative TB which is common among PLHIVs [4]. TB treatment failure diagnosis poses a greater challenge because it requires specialized laboratories to isolate M. tuberculosis. These facilities are not widely available in most resource poor countries [33]and therefore not available for routine screening of HIV infected patients. This leaves a large proportion of patients who are HIV infected and at high risk of TB treatment failure rates compared to self-administered therapy [5]. Poor compliance has been implicated in the development of TB treatment failure [7.5,6]. Although the proportion of patients on DOT as recorded in the treatment register was higher than reported during patient interviews, DOT remained protective and an independent predictor of TB treatment failure (OR = 0.28; P < 0.0001). These findings mirror those of a case-control study conducted in Hong Kong [3]. DOT plays a crucial role in preventing the development of drug resistance by ensuring that patients ingest the correct dose of TB medication regularly and for the prescribed duration.

The current TB pandemic is intertwined with the HIV/AIDS pandemic. HIV-positive status has consistently been linked to poor outcomes of TB treatment in various studies, including a case-control study in Peru [34]and across four European countries [3], as well as retrospective studies in South Africa and the USA[7]. Across these studies, HIV positivity consistently increased the risk of TB treatment failure. However, a descriptive study in India reported a weaker association with HIV [9]. Studies conducted in Africa, including those in KwaZulu Natal, South Africa [7], and Mozambique [12], identified HIV as a major risk factor for TB treatment failure. These findings align with global TB treatment failure surveillance data, indicating a convergence of TB treatment failure and HIV/AIDS. Some studies suggest that HIV infection may prompt the development of rifampicin resistance [5,8,9], while others found no demonstrated association between HIV infection and TB treatment failure [5].

Multivariate analysis in our current study revealed that a significantly larger proportion of controls were HIV seropositive compared to cases (P = 0.0253). When interpreting these results, it’s crucial to consider factors surrounding TB treatment failure surveillance, epidemiology, and control in Kenya, which may substantially differ from other countries. These factors may accelerate TB disease progression among HIV-infected patients, selectively impact the survival and transmission of TB treatment failure among HIV-seronegative individuals.

Many African countries, including Kenya, are reported to underreport cases of TB treatment failure [7]. Additionally, Kenya has a high prevalence of HIV-related TB, which is challenging to diagnose due to atypical presentations and the lack of appropriate tests to detect smear-negative TB, common among people living with HIV [3]. Diagnosis of TB treatment failure presents a significant challenge, as it requires specialized laboratories to isolate M. tuberculosis, which are not widely available in most resource-poor countries [4], thereby limiting routine screening for HIV-infected patients at high risk of TB treatment failure unscreened.

The approach to treating smear-positive TB cases typically involves allowing the treatment to proceed, with suspicion of TB treatment failure only arising in the second month if there is evidence of delayed sputum conversion. However, this protocol may not apply to HIV-positive TB patients, who may present with smear-negative pulmonary tuberculosis. HIV-infected individuals with TB treatment failure often experience exceptionally high mortality rates [4]. Progression of infection in these patients can be rapid, with death occurring within four weeks of diagnosis, a timeframe shorter than that required for traditional culture-based diagnosis of TB treatment failure [2–4,8]. Consequently, many patients with TB treatment failure may succumb before being suspected or diagnosed. In Peru, for example, a third of HIV-positive patients diagnosed with TB treatment failure died in less time than it typically takes to complete susceptibility studies [3]. The findings from our study also indicate possible transmission of TB treatment failure among HIV-negative individuals, which could partly explain the higher proportion of HIV-seronegative individuals among the cases. A combination of these factors may lead to selective diagnosis of TB treatment failure in HIV-negative patients and selective survival of HIV-negative TB treatment failure patients, thus accounting for the observed association. Further studies are warranted to elucidate the association between HIV and TB treatment failure in Kenya.

Bacillary load on sputum smear microscopy often correlates with the severity of disease. Patients with cavitary pulmonary tuberculosis and lung damage typically exhibit a high bacillary load, which is associated with an increased risk of random mutations leading to drug resistance [14]. In our study, a bacillary load of 2+ or higher at the diagnosis of the last episode of tuberculosis was associated with TB treatment failure (P = 0.002). However, it was not identified as an independent predictor of TB treatment failure. These findings align with those of a prospective study in India [13]. High bacillary load has been shown to correlate with the degree of infectiousness and the risk of developing drug resistance [14].

In Eastern Africa, the Bacillus Calmette-Guérin (BCG) vaccine is routinely administered as part of immunization programs. The presence of a BCG scar is considered evidence of BCG vaccination. BCG has been found to provide varying levels of protection, ranging from none to up to 80% among children, with a consistent decline in protective efficacy with increasing age, eventually offering no protection in adults. In Kenya, a case-control study of risk factors for tuberculosis among prisoners revealed a protective association between the presence of a BCG scar and active tuberculosis [19]

In our study, the presence of a BCG scar appeared to provide protection against TB treatment failure in the bivariate analysis (P = 0.05), although it did not emerge as an independent predictor of TB treatment failure. This finding appears to challenge the hypothesis that prolonged use of the BCG vaccine in a population may selectively eliminate Beijing genotypes of M. tuberculosis, which are associated with TB outbreaks and an increased risk of treatment failure [2]. The Beijing family genotypes are characterized by significant pathogenic features such as high virulence, multi-drug resistance, and increased susceptibility to exogenous reinfection.

While there have been relatively few studies investigating the effect of BCG vaccination on chronic excretion of M. tuberculosis, a study examining tuberculosis transmission among close contacts of TB treatment failure cases found a protective association that remained significant even after controlling for age, sex, race, purified protein derivative (PPD) status, and isoniazid prophylaxis [7]. Another study indicated that BCG conferred protection for 50–60 years [9,10]. Given the emergence of drug-resistant TB strains in many regions, there is a growing recommendation for BCG vaccination among adults with prolonged exposure to MDR-TB, particularly in settings where TB prevalence is high and where BCG has been shown to offer protection in adults [3,7].

### Limitations of the study

Confirmation of diagnosis among the controls relied on sputum smear microscopy rather than culture and drug susceptibility testing (DST) to definitively rule out TB treatment failure. This could potentially lead to misclassification of controls who may have actually experienced treatment failure. However, it’s important to note that the proportion of TB treatment failure cases in Nairobi County is relatively small. Additionally, the observed clinical and bacteriological response among the controls is assumed to be attributed to the efficacy of first-line anti-tuberculosis drugs rather than the natural healing process of tuberculosis, which could occur in approximately 50% of patients if left untreated over a two-year period. It’s unlikely that this misclassification would significantly bias the results, as it would tend towards null findings and therefore not explain any significant differences observed in the study.

Establishing a cause-effect relationship is challenging in this study, as both the exposure and outcome had already occurred at the time of conducting the study. Cases had a longer mean duration of illness compared to controls and had undergone multiple unsuccessful TB treatments. Consequently, they may have been more likely to recall events surrounding their illness compared to controls. Efforts were made to mitigate this bias by focusing on exposures that do not change rapidly over time. It was assumed that cases were less likely to recall exposures compared to controls, so any existing recall bias would likely affect both groups equally, potentially underestimating the strength of association.

As a case-control study, the selection of study sites was based on the presence of treatment failure cases rather than a random selection of facilities offering TB care. This may have led to varying levels of representativeness across different areas, with some areas having more selected facilities than others. However, efforts were made to include all available cases, while controls were randomly selected within the chosen facilities to mitigate this limitation

## Conclusions

Clinical factors derived from treatment records and patient interviews, including previous TB treatment, positive sputum smear at two months, poor adherence to treatment, DOT program was found to be protective against TB treatment failure. A potential protective association was observed between TB treatment failure and recorded HIV-positive sero status. Based on these findings, clinical factors are associated with TB treatment failure in Nairobi County, some of

which can be modified through public health interventions. Therefore, the null hypothesis is rejected.

This case-control study suggests the following recommendations: Strengthen management of drug-susceptible TB cases by enhancing DOT and improving recording practices to prevent treatment failure across all population groups. Expand TB treatment failure surveillance to include all patients with a history of previous TB treatment, those with positive sputum smear at 2 months of treatment, infectious TB cases (sputum smear-positive), contacts of known TB cases, and all HIV-positive TB patients. Strengthen contact tracing, screening, and documentation of TB treatment failure cases and Conduct further studies to elucidate the association between HIV and TB treatment failure.
